# Annotations

**Published:** 1909-07-03

**Authors:** 


### ANNOTATIONS.

### The Darwin Centenary.

THOUGH Charles Darwin was not a member of the medical profession, there is assuredly no branch of science, apart from those of biology and geology in which he was himself so expert, in which the effect of Darwin's lifework has been so far reaching as in that of medicine. Inductive philosophy was not, of course, unknown or even unfamiliar before the appearance of the *Origin of Species* fifty years ago; it would be strange if in the motherland of Bacon such a thing could ever happen. But there is no doubt that as far as therapeutic science was concerned, it was insufficiently appreciated. The patient accumulation of every fact which could bear upon a given problem, the laborious testing of every link in the chain of argument, in fact all the philosophic qualities displayed in Charles Darwin's works in so conspicuous a degree, have consciously or unconsciously influenced all those workers whose researches have revolutionised medicine and surgery in the last half-century. Pasteur, Lister, Koch, and many others, are all disciples of Darwin, in that his example and his methods are the familiar patterns of what those of a scientist should be. Physiology and even perhaps pathology, as well as zoology and botany, have been profoundly influenced by the theory of evolution; comparative anatomy is practically a product of Darwin's conceptions, and the whole science of human anatomy, including embryology, has been illuminated by a flood of light from the same source. No medical student can complete—indeed, he can scarcely even commence—his studies without being brought constantly into contact with Darwin's work or its results; and, if it would be too much to assert, that every one of them has read one or more of the classic treatises in which that work was set before the world, this is at least, we imagine, true of the majority. Beyond and above his eminence as a scientist, Darwin was also the very type of an English gentleman. On both accounts it is well that Medicine should have been represented at the Cambridge celebrations of the centenary of his birth: Charles Darwin's memory is indeed well worthy to be kept green by our profession.

### Birthday Honours and the Medical Profession.

FEW will deny that the medical profession as a whole is accustomed to look forward, when it thinks about the matter at all, to the periodical announcements of titles and other marks of Royal favour, with interest and a sense of pleasurable anticipation. And it is hard to recall any publication of Birthday or New Year's Honours which hurt the feelings of the medical world, or called forth protest or ridicule within its ranks. Fortunately, it is not yet a popular prank with idle mischief-makers to analyse these rolls of honour, with a view to invidious contrasts between the distinctions apportioned to the several professions and branches of public service. The medical Birthday Honours for 1909 are distributed, wisely and with a due sense of proportion, amongst men who have done good work in diverse regions of medical activity. These men include clinicians, scientific investigators, public servants in various departments at home and abroad, and representatives of governing medical corporations. All have done solid work on behalf of the progress and honour of medicine; and all have achieved something for the benefit of the community. The many medical men who have worked under them, or profited by the published results of their labours, will read with special pleasure of the elevation of Sir Dyce Duckworth to a baronetcy; the conferment of a similar honour upon Mr. Henry Morris, the distinguished president of the English College of Surgeons; and the knighthood of Lieut. Colonel Leishman, who has done so much to advance the scientific reputation of the Royal Army Medical Corps. The promotion of Surgeon-General L. D. Spencer to be K.C.B. is a tribute to many years of good work in the Indian medical service; while Professor W. J. R. Simpson's efforts towards plague prevention, and on behalf of Colonial hygiene generally, have earned him the C.M.G. Another knighthood, though not of a medical man, will in the future gain more and more appreciation from our profession, as the pioneer work in the field of Eugenics of Mr. Francis Galton, the cousin of Darwin, bears more and more fruit. Military medical service at home has gained the C.B. for Surgeon-General H. R. Whitehead and Colonel de Burgh Birch; while, in India, Lieut.-Colonel R. N. Campbell, I.M.S., and Mr. Edgar Thurston have each received the C.I.E.—the one for military service and the other for ethnological research—and a native practitioner, Dr. Nariman, has been awarded the Kaisar-i-Hind medal.

### Illegal Operations.

IN view of the very great difficulty which attends the detection and conviction of those who, whether medically qualified or not, perform illegal operations, it is satisfactory to note the examplary punishment meted out on June 25 at the Central Criminal Court to William Arthur Jones, "calling himself a doctor," who was sent to penal servitude for ten years for this offence. This man was struck off the Medical Register in 1902 for practices of a similar kind, and it is clear that society is well rid of him for a considerable space of time. On the same occasion sentence of three months was passed upon the man who had suggested to the patient the operation in question, and arranged for its performance; in view of the fact that the woman was a domestic servant in his house, this prisoner must be regarded as extremely lucky to escape with so comparatively light a punishment. It does not appear from the brief newspaper report whether any extenuating circumstances existed with regard to this particular prisoner; but on general grounds the person who suggests and procures such crimes should be held but little less culpable than he who actually carries them out.

